# Impacts of non-nutritive sweeteners on the human microbiome

**DOI:** 10.1097/IN9.0000000000000060

**Published:** 2025-04-24

**Authors:** Katti R. Crakes, Lauren Questell, Subah Soni, Jotham Suez

**Affiliations:** 1 W. Harry Feinstone Department of Molecular Microbiology and Immunology, Johns Hopkins Bloomberg School of Public Health, Baltimore, MD, USA

**Keywords:** non-nutritive sweeteners, artificial sweeteners, low-calorie sweeteners, microbiome, microbiota, metabolic syndrome, diabetes, obesity, diet

## Abstract

Replacing sugar with non-nutritive sweeteners (NNS) is a common dietary strategy for reducing the caloric content and glycemic index of foods and beverages. However, the efficacy of this strategy in preventing and managing metabolic syndrome and its associated comorbidities remains uncertain. Human cohort studies suggest that NNS contribute to, rather than prevent, metabolic syndrome, whereas randomized controlled trials yield heterogeneous outcomes, ranging from beneficial to detrimental impacts on cardiometabolic health. The World Health Organization recently issued a conditional recommendation against using NNS, citing the need for additional evidence causally linking sweeteners to health effects. One proposed mechanism through which NNS induce metabolic derangements is through disruption of the gut microbiome, a link strongly supported by evidence in preclinical models. This review summarizes the evidence for similar effects in interventional and observational trials in humans. The limited available data highlight heterogeneity between trials, as some, but not all, find NNS consumption associated with microbiome modulation as well as metabolic effects independent of sweetener type. In other trials, the lack of microbiome changes coincides with the absence of metabolic effects. We discuss the hypothesis that the impacts of NNS on health are personalized and microbiome dependent. Thus, a precision nutrition approach may help resolve the conflicting reports regarding NNS impacts on the microbiome and health. This review also discusses additional factors contributing to study heterogeneity that should be addressed in future clinical trials to clarify the relationship between NNS, the microbiome, and health to better inform dietary guidelines and public health policies.

## 1. Non-nutritive sweeteners and human health – where are we now?

The global prevalence of metabolic syndrome (MetS), overweight, and obesity is at an all-time high. MetS is defined by having at least three of the following conditions: hyperglycemia, hypertension, increased waist circumference, low high-density lipoprotein (HDL) cholesterol, and elevated fasting triglycerides. These factors collectively increase the risk of developing diabetes, heart disease, stroke, and several types of cancer ^[1]^. Estimates on the global prevalence of MetS range from 12.5% to 34% depending on the diagnostic criteria used ^[2]^, and 38% of adults are classified as overweight ^[3]^. MetS is a global concern, yet its prevalence is notably higher in developed countries. For example, in the United States, 70% of adults are classified as overweight ^[4]^, and one in three adults is affected by MetS ^[5]^.

Against this backdrop, the impact of non-nutritive sweeteners (NNS) on human health has become a subject of intense debate. NNS confer a sweet taste to foods and beverages with little to no added calories, and their consumption does not elicit a post-prandial glucose response, making them appealing to consumers focused on weight management and blood sugar control. The United States Food and Drug Administration (FDA) has approved the following non-nutritive artificial sweeteners as safe for use through its food additive approval process: saccharin, sucralose, aspartame, neotame, advantame, and acesulfame-potassium (Ace-K). Additionally, the plant-based natural NNS stevia, allulose, and monk fruit are classified as Generally Recognized as Safe by the FDA, allowing their use in food products. Another artificial non-nutritive sweetener, cyclamate, is not approved for use in the United States but is common elsewhere ^[6]^. Concerns regarding the health impacts of sugar and caloric sweeteners are shifting consumer preferences towards products sweetened with NNS or a mix of both caloric and non-caloric sweeteners ^[7,8]^, and this trend is expected to continuously grow globally ^[9]^. Despite their immense popularity and potential benefits over caloric sweeteners, the increased prevalence of NNS-containing products and their consumption is paradoxically accompanied by an increase, rather than a decrease, in the incidence of MetS ^[10]^.

The considerable body of research on the impacts of NNS on human health is comprised of observational cohort trials and interventional randomized controlled trials (RCTs). The latter are considered the gold standard for determining causality due to randomization, which minimizes bias and confounding. Cohort trials, while more prone to confounding, allow for the study of long-term outcomes in real-world settings and are often more feasible for studying large populations and outcomes not seen in short-term exposures that are common in RCTs. The World Health Organization (WHO) has recently conducted a systematic review and meta-analysis (*N* = 283 studies) to address the growing concerns regarding the health impacts of NNS, including both observational trials and RCTs ^[11]^. In RCTs, consumption of NNS vs caloric sweeteners was associated with modest short-term weight loss (−0.14 kg/m², low certainty of evidence) and no significant (beneficial or detrimental) impact on body composition, blood pressure, blood lipids, and markers of type 2 diabetes. In long-term observational trials, NNS consumption was associated with a higher incidence of obesity, type 2 diabetes, cardiovascular disease, preterm birth, and all-cause mortality. Based on these findings, the WHO recommended that NNS should not be used as a means of achieving weight control or reducing the risk of non-communicable diseases. In labeling the recommendation as “conditional”, the WHO cited the low certainty of the overall evidence for both beneficial and undesirable effects and the challenge of causally linking NNS consumption to disease outcomes.

In pursuit of causal evidence and mechanistic insights, many researchers model the impacts of NNS on metabolic health in laboratory animals. Most, but not all, animal studies demonstrate a detrimental impact of NNS on metabolic health. These studies also identified the gut microbiome as a modulator of NNS impacts on the host ^[12]^, demonstrating that various NNS alter the composition of naturally occurring microorganisms in the gut and/or their metabolic output, resulting in configurations that may adversely impact metabolic health.

## 2. Diet, the gut microbiome, and metabolic health

The gut microbiome plays an important role in the development and homeostatic function of virtually all aspects of human physiology, including cardiometabolic health ^[13]^. Gut bacteria and viruses have been demonstrated to play a causative role in the pathogenesis of MetS and its sequelae, including obesity, diabetes, metabolic liver disease, atherosclerosis, and ischemic vascular diseases ^[14]^. This is mediated through multiple mechanisms that are the topic of extensive research, including the secretion and modulation of host-targeting metabolites (eg, short-chain fatty acids [SCFA], branched-chain amino acids, secondary bile acids, trimethylamine *N*-oxide, indole and its derivatives), modulation of systemic and localized immune responses, production and modulation of neurotransmitters (eg, serotonin, catecholamine, γ-aminobutyric acid, dopamine, histamine), signaling through the gut–brain axis to impact feeding behavior, and impacts on intestinal barrier function, among others ^[14,15]^.

The microbiome also modulates cardiometabolic health through its crosstalk with the host’s diet. The nutrient content and their quantity in the diet, as well as the timing of food intake, can all impact the microbiome through direct (eg, microbial utilization of nutrients) and indirect (eg, immunomodulatory) mechanisms ^[16]^, leading to dietary pattern-specific microbial configurations and downstream impacts on health ^[17]^. Both macronutrients (dietary fiber, fat, protein) and those found in lower quantities (eg, polyphenols) drive the interaction between diet and the microbiome. In addition to naturally occurring nutrients, the microbiome can also mediate the health impacts of xenobiotics and man-made food additives, including those commonly found in ultra-processed foods (UPFs) ^[18]^. This food category (NOVA group 4) refers to industrial formulations made mostly or entirely from substances extracted from food (oils, fats, sugar, starch, protein), derived from food constituents, or synthesized in laboratories from food substrates or other organic sources, such as sweeteners, flavors, texturizers, colors, and preservatives. Diets high in UPFs have been associated in cohort trials with detrimental impacts on overall health, particularly metabolic health ^[19,20]^. These diets have been shown to significantly alter the gut microbiome in both humans ^[19,21]^ and preclinical models ^[22]^, which has been directly linked to adverse metabolic outcomes ^[19,21]^. Specific components of ultra-processed diets, such as emulsifiers, colorants, and preservatives, have been identified as key contributors to negative health consequences. Emulsifiers, which are added to improve texture and extend shelf life, have been shown to promote bacterial translocation across the gut epithelium and increase inflammation, leading to metabolic disturbances ^[23–25]^. Synthetic colorants, used to enhance the visual appeal of foods, have been implicated in triggering intestinal inflammation ^[26–28]^. Preservatives designed to prolong the shelf life of food products can suppress the growth of beneficial bacteria, contributing to dysbiosis and metabolic derangements ^[29–31]^. Collectively, the extensive crosstalk between the microbiome and dietary components, including man-made additives, provides a robust rationale for examining the interaction between NNS and the microbiome in the context of cardiometabolic health.

## 3. Modulation of the human microbiome by NNS

Most of the evidence for NNS impacts on the gut microbiome stems from preclinical feeding trials. We have recently reviewed the available literature and reported that the majority of animal studies (but not all) observed alterations in the gut microbiome induced by NNS feeding, regardless of the type, dose, administration mode, or formulation of the sweetener, the animal species, age, or sex, or the microbiome profiling method ^[12]^. For the most part, these preclinical studies have further associated microbiome alterations with a detrimental impact on metabolic health, and three studies have causally linked the two by showing that a microbiome from NNS-exposed animals is sufficient to promote weight gain and/or glucose intolerance in NNS-naïve germ-free (GF) recipients ^[32–34]^. While preclinical studies are useful for understanding potential mechanisms, it is important to note that health effects observed in animals do not always translate to humans. These studies also do not account for the complexity of human dietary components, which can have profound effects on the gut microbiome. Nonetheless, the impact of some diets and nutrients on the murine microbiome can be replicated in human gut communities ^[35]^, and dietary intervention studies in mice have been successfully translated to human clinical trials ^[36]^, supporting the importance of examining whether NNS-induced dysbiosis observed in rodents occurs in humans as well.

Compared with animal models, considerably fewer studies have examined the impact of NNS on the human microbiome. Focusing on NNS of artificial (acesulfame-potassium, aspartame, cyclamate, neotame, saccharin, and sucralose) or natural/plant-based (stevia, allulose, and monk fruit) sources, we found a total of nine interventional studies with 14 NNS intervention arms as follows: sucralose (4 arms) ^[33,37–39]^, saccharin (3) ^[32,33,40]^, stevia (3) ^[33,41,42]^, aspartame (2) ^[33,39]^, and an Ace-K and sucralose blend (2) ^[43]^. We did not find studies examining the impact of cyclamate, neotame, advantame, monk fruit, allulose, or Ace-K (not as part of a blend) on the human microbiome. While cyclamate is not approved for consumption in the United States and is losing popularity in Europe, it is highly consumed (by volume) in other parts of the world ^[44]^. Furthermore, consumers increasingly favor natural/plant-based NNS over artificial ones ^[8,45]^. These global consumption trends should be considered when designing additional human trials on NNS, microbiome, and health.

Overall, of the 14 aforementioned experimental arms, eight reported a significant impact of NNS on the human gut microbiome ^[32,33,37,43]^, one reported limited impact ^[41]^, and five did not observe a significant effect ^[38–40,42]^ (Table 1). Only saccharin, sucralose, and stevia were tested in more than two trials, and for all three sweeteners, there was no consensus between the trials regarding their impacts on the microbiome. Thus, based on these studies, the type of sweetener used is not the reason for the heterogeneity between trials. Noteworthy, there was generally an agreement between the impacts of NNS on metabolic health and the microbiome; of the eight arms that reported a significant impact on the microbiome, three also observed significant disruptions to glucose homeostasis ^[12,37]^, three found a correlation between the microbiome and metabolic readouts ^[32,33]^, one reported changes in inflammatory pathways expression in subcutaneous adipose tissue ^[43,46]^, and one did not report any metabolic readouts beyond weight (which was not significantly impacted) ^[43]^. In contrast, none of the null microbiome studies found any significant impacts on metabolic parameters (Table 1). Thus, the impact of NNS on metabolic health may depend on the extent to which the sweeteners alter the microbiome and could potentially be used to predict personalized responses to NNS.

**Table 1:** Summary of interventional trials investigating the effects of non-nutritive sweeteners (NNS) on the human fecal microbiome and metabolic outcomes.

Study	Country	Size (% F)	NNS	Formulation	% ADI^*^	Duration of exposure	Profiling method	Microbiome changes	Metabolic changes
Suez et al ^[33]^	Israel	*N =* 20 (45%)	Saccharin	Commercial sachets (+D)	16%	2 weeks	MG	Yes	Yes
Suez et al ^[32]^	Israel	*N =* 7 (29%)	Saccharin	Commercial sachets (+D)	11%	1 week	16S	Yes^†^	Yes^†^
Suez et al ^[33]^	Israel	*N =* 20 (65%)	Sucralose	Commercial sachets (+D)	27%	2 weeks	MG	Yes	Yes
Méndez-García et al ^[37]^	Mexico	*N =* 20 (70%)	Sucralose	Pure sucralose in sterile water	13%	10 weeks	qPCR	Yes	Yes
Sylvetsky et al ^[43]^	United States	*N =* 8 (50%)	Sucralose + Ace-K	Diet Rite Cola™	54% Sucralose, 11% Ace-K	1 week	MG	Yes	NR
Sylvetsky et al ^[43]^	United States	*N =* 8 (100%)	Sucralose + Ace-K	Diet Pepsi™	19% Sucralose, 11% Ace-K	8 weeks	MG	Yes	Limited^‡^
Suez et al ^[33]^	Israel	*N =* 20 (50%)	Aspartame	Commercial sachets (+D)	6%	2 weeks	MG	Yes	Yes^†^
Suez et al ^[33]^	Israel	*N* = 20 (50%)	Stevia	Commercial sachets (+D)	60%	2 weeks	MG	Yes	Yes^†^
Singh et al ^[41]^	United Kingdom	*N =* 14 (79%)	Stevia	SteviaClear® Sweet Drops®	NR	12 weeks	16S	Limited	No
Serrano et al ^[40]^	United States	*N =* 13 (69%)	Saccharin	Sodium saccharin capsule	36%	2 weeks	16S	No	No
Thomson et al ^[38]^	Chile	*N =* 17 (0%)	Sucralose	Sucralose capsule (+CC)	208%	1 week	16S	No	No
Ahmad et al ^[39]^	Canada	*N =* 17 (59%)	Sucralose	Pure sucralose in water (+CA, LE)	36%	2 weeks	16S	No	No
Ahmad et al ^[39]^	Canada	*N =* 17 (59%)	Aspartame	Pure aspartame in water (+CA, LE)	11%	2 weeks	16S	No	No
Kwok et al ^[42]^	Canada	*N =* 27 (59%)	Stevia	Steviol glycoside beverage (+CA, PC, NC, NF)	25%	4 weeks	MG	No	No

The table presents data from various studies, categorized by country, cohort size (including percentage of female participants), type of NNS, formulation details, duration of exposure, profiling method used for microbiome analysis, observed microbiome changes, and reported metabolic outcomes. The data are based on information reported by the authors in the studies referenced. Compilation of clinical studies was retrieved from https://pubmed.ncbi.nlm.nih.gov/ using query terms “Human microbiome OR Human microbiota” AND 1. “Artificial sweeteners OR Non-nutritive sweeteners” and 2. “Saccharin OR Sucralose OR Aspartame OR Acesulfame-Potassium OR Ace-K OR Neotame OR Advantame OR Stevia.” Ace-K, acesulfame-potassium; ADI, acceptable daily intake; CA, citric acid; CC, calcium carbonate; D, dextrose; F, female; LE, lemon extract; MG, metagenomics; NC, natural color; NF, natural flavor; NNS, non-nutritive sweetener; NR, not reported; PC, potassium citrate.

* The supplemented dose is summarized as % of the acceptable daily intake (ADI) for an individual weighing 75 kg based on the US Food and Drug Administration (FDA) definition. Doses for studies with stevia likely encompass different types, combinations, and ratios of steviol glycosides.

† In responders.

‡ Altered adipose transcriptomic signatures.

There were multiple methodological differences between the nine trials that might underlie the heterogeneity in outcomes. Most trials were short (1–2 weeks of exposure), but some trials were longer (4–12 weeks). The supplemented dose ranged from 6% of the acceptable daily intake to 208%. The number of participants in the exposure arms also varied (*N* = 7–27, average 16.6). While one study only recruited males ^[38]^ and another only recruited females ^[43]^, the representation of males and females was comparable between trials (average 56% females). The small number of published studies on the topic does not allow for drawing definitive conclusions regarding the contribution of each variable to the outcome, and more data are critically needed. However, exposure duration and dose, as well as the cohort size and the participant’s sex, are less likely to be the major drivers of the heterogeneity between the trials’ outcomes, as they are not qualitatively different between the null studies and those that reported a significant effect (Table 1). In contrast, baseline NNS exposure, supplementation mode, and the microbiome profiling method (16S amplicon sequencing vs shotgun metagenomics) may play a role in explaining the differences between the trials’ outcomes.

### 3.1 Inclusion and exclusion criteria (baseline NNS exposure)

All but one study ^[38]^ reported excluding habitual or frequent consumers of NNS-containing products from participation. Of these, three studies (seven arms) reported using a detailed multi-item food frequency questionnaire to exclude any consumers of NNS-containing products ^[32,33,43]^. Three studies (four arms) included participants if habitual NNS consumption was lower than an indicated threshold ^[40–42]^, one sucralose supplementation study excluded habitual sucralose consumers and did not provide additional details on this criterion ^[37]^, and one study did not detail how NNS consumers were identified and excluded ^[39]^. Interestingly, of the six arms that reported limited or no significant impact of NNS on the human microbiome, four included current NNS consumers ^[40–42]^ and one did not specify whether NNS consumers were excluded ^[38]^. Therefore, it is possible that baseline NNS consumption was higher in trials reporting null results, especially considering that inadvertent NNS exposure may be common ^[47,48]^. When attempting to identify dietary modulators of MetS, isolating the de novo impacts of NNS on the gut microbiome and metabolic health might require minimal pre-exposure levels. Importantly, this hypothesis has yet to be experimentally validated, and the required threshold, if one exists, is unclear.

### 3.2 Microbiome profiling method

Characterizing microbial communities using shotgun metagenomics is becoming increasingly popular over 16S rRNA amplicon sequencing, as the former allows identifying bacteria at a higher (more specific) taxonomic resolution and provides insights into enriched microbial functions and pathways. Notably, six of eight arms that reported a significant impact of NNS on the microbiome utilized shotgun metagenomics, compared with five of six null and limited impact arms that used 16S sequencing (Table 1). Noteworthy, the null trial that used metagenomics did not examine the impact of NNS on the microbiome-encoded functions. In Suez et al ^[33]^, the authors reported more significant impacts of four NNS on the microbiome functions than on its composition. The human microbiome composition is highly heterogeneous and individualized, but many core functions are redundant, meaning that different bacteria perform similar functions ^[49]^. Thus, examining the impact of an intervention on encoded functions can be more insightful, as interindividual differences might mask a compositional signal.

### 3.3 Supplementation method

In real-life scenarios, NNS are often not consumed in their pure form but are rather added to other ingredients. For example, sachets containing NNS often use glucose or other carbohydrates as bulking agents. It has been hypothesized that consuming NNS with carbohydrates is more detrimental to metabolic health than consuming NNS on their own ^[50]^. Whether this also applies to NNS’ ability to impact the microbiome is currently unknown. Notably, in all the studies that did not find an impact of NNS on the microbiome, the NNS were supplemented without an added carbohydrate moiety, either as pills or as carbohydrate-free beverages. In contrast, five of eight arms that reported a significant impact of NNS on the microbiome provided the sweeteners as commercially available sachets containing glucose as a bulking agent (Table 1). In one of the studies (four arms), a glucose-supplemented group was included to confirm that the observed impacts were not attributed to glucose ^[33]^.

Observational trials play an important role in understanding the health effects of NNS ^[11]^. While such trials cannot provide a direct causal link between NNS consumption and health outcomes, observational trials are often considerably longer than RCTs, providing an opportunity to observe outcomes not seen in short-term exposure or are ethically unfeasible (such as diabetes complications, cancer, dementia and stroke, and all-cause mortality) ^[51–53]^. Furthermore, these trials often monitor much larger cohorts. As the nature and magnitude of health impacts can vary between different types of NNS, large enough trials may be sufficiently powered to explore sweetener-specific associations ^[53]^. Furthermore, large cohorts can facilitate the stratification of participants according to risk factors and the identification of risk modulators. Noteworthy, cohort trials commonly adjust for multiple covariates and perform sensitivity analyses, which are important for addressing reverse causation, namely, that a higher intake of NNS in individuals with metabolic (and other) derangements is not the cause of these health conditions but rather reflects pre-existing poorer metabolic health at baseline, which drives these individuals to consume NNS. Indeed, the association between NNS intake and detrimental health outcomes in cohort trials is robust to adjustment for multiple covariates, most commonly age, sex, smoking, alcohol intake, habitual diet (primarily total energy and sugar intake), body mass index (BMI), and disease risk ^[11]^. In many of these trials, the association is significant even after sensitivity analyses that exclude cases in the first few years of follow-up. Thus, it is less likely that reverse causation is the sole explanation for the association between NNS intake and detrimental health outcomes. This is further strengthened by a considerable number of studies linking NNS intake to metabolic derangements in rodents ^[12]^, including several that show that fecal microbiome transplantation from NNS-exposed humans or rodents is sufficient to impair metabolic health in NNS-naïve animals ^[32–34]^.

Considering the limited interventional studies examining the impact of NNS on the human microbiome, we broadened our search and found seven relevant observational studies ^[32,54–59]^. All trials used food frequency questionnaires (FFQs) to identify NNS consumers and found significant correlations between NNS intake and an impact on the microbiome (profiled through 16S sequencing) (Table 2), although it should be noted that FFQs are prone to recall bias and inaccuracies in diet reporting. Beyond that, trials varied in their design. The largest trial (*N* = 381) recruited adults without diabetes and used long-term FFQs to identify high consumers of NNS (*N* = 40) and abstainers. Consumption of NNS was associated with multiple markers of MetS, including higher glycosylated hemoglobin (% HbA1c) and elevated liver enzymes. In a randomly selected subcohort (*N* = 172), NNS consumption was associated with multiple taxonomic signatures independent of BMI ^[32]^. Another cross-sectional study also found correlations between NNS intake and several gut microbial taxa in individuals with morbid obesity (*N* = 89) ^[58]^. While observational trials often aggregate the consumption of any NNS as one group, a cross-sectional study by Frankenfeld et al (*N* = 31) identified consumers of either aspartame or Ace-K. The microbiome of consumers of each sweetener significantly differed from that of non-consumers ^[56]^. Another study of participants without diabetes separated consumers of aspartame and consumers of non-aspartame non-sugars and compared their fecal (*N* = 40) and duodenal (*N* = 99) microbiome to that of control individuals. The authors reported significant differences in the relative abundances of multiple taxa in both stool and duodenal samples. Notably, the microbial signature of NNS exposure was different and, in some cases, even opposite between these two sites ^[57]^. Considering the growing recognition that stool samples do not accurately reflect the gut microbiome in situ ^[60,61]^ and the important metabolic, endocrine, and immune functions occurring in the small intestine, further exploration of NNS impacts on the microbial community in this gut region could help shed light on the mechanisms through which sweeteners impact our health. Two additional diet-microbiome studies (*N* = 75; *N* = 98) identified taxonomic signatures associated with the consumption of specific NNS ^[54,55]^.

**Table 2:** Summary of observational trials assessing the impact of non-nutritive sweeteners (NNS) on the human fecal microbiome.

Study	Country	Size (% F)	NNS	Profiling method	Microbiome changes
Tang et al ^[54]^	United States	*N =* 136 (63%^*^)	Sucralose	16S	Yes
Wu et al ^[55]^	United States	*N =* 98 (56%)	Sucralose	16S	Yes
Frankenfeld 2015 ^[56]^	United States	*N =* 7 (65%^*^)	Ace-K	16S	Yes
Wu et al ^[55]^	United States	*N =* 98 (56%)	Ace-K	16S	Yes
Frankenfeld et al ^[56]^	United States	*N =* 7 (65%^*^)	Aspartame	16S	Yes
Hosseini et al ^[57]^	United States	*N =* 4 (75%)	Aspartame	16S	Yes
Tang et al ^[54]^	United States	*N =* 136 (63%^*^)	Aspartame	16S	Yes
Wu et al ^[55]^	United States	*N =* 98 (56%)	Aspartame	16S	Yes
Farup et al ^[58]^	Norway	*N =* 89 (84*%*)	Any NNS	16S	Yes
Hosseini et al ^[57]^	United States	*N =* 11 (73%)	Any NNS	16S	Yes
Laforest-Lapointe et al ^[59]^	Canada	*N =* 50 (46%)	Any NNS^†^	16S	Yes
Suez et al ^[32]^	Israel	*N* = 172 (56%^*^)	Any NNS	16S	Yes

The table summarizes findings from studies categorized by study size (including the percentage of female participants), type of NNS assessed, microbiome profiling method (16S rRNA sequencing), and observed changes in the microbiome.

* Percentage of females reported within the entire cohort, not limited to the subset used for microbiome profiling.

† In utero maternal exposure.

In contrast to the aforementioned trials examining the impact of direct NNS consumption, Laforest-Lapointe et al ^[59]^ found correlations between high maternal NNS consumption and a higher infant BMI, as well as impacts on the infant microbiome structure and urinary metabolites (*N* = 100). The mechanism through which maternal NNS consumption impacts the infant microbiome is unclear. Some NNS have been found in breast milk ^[62]^, which could be a possible route of exposure. Alternatively, the maternal microbiome, altered by NNS, could be acquired by the infant during birth and early life ^[59]^, though additional research is required.

**Figure 1.**
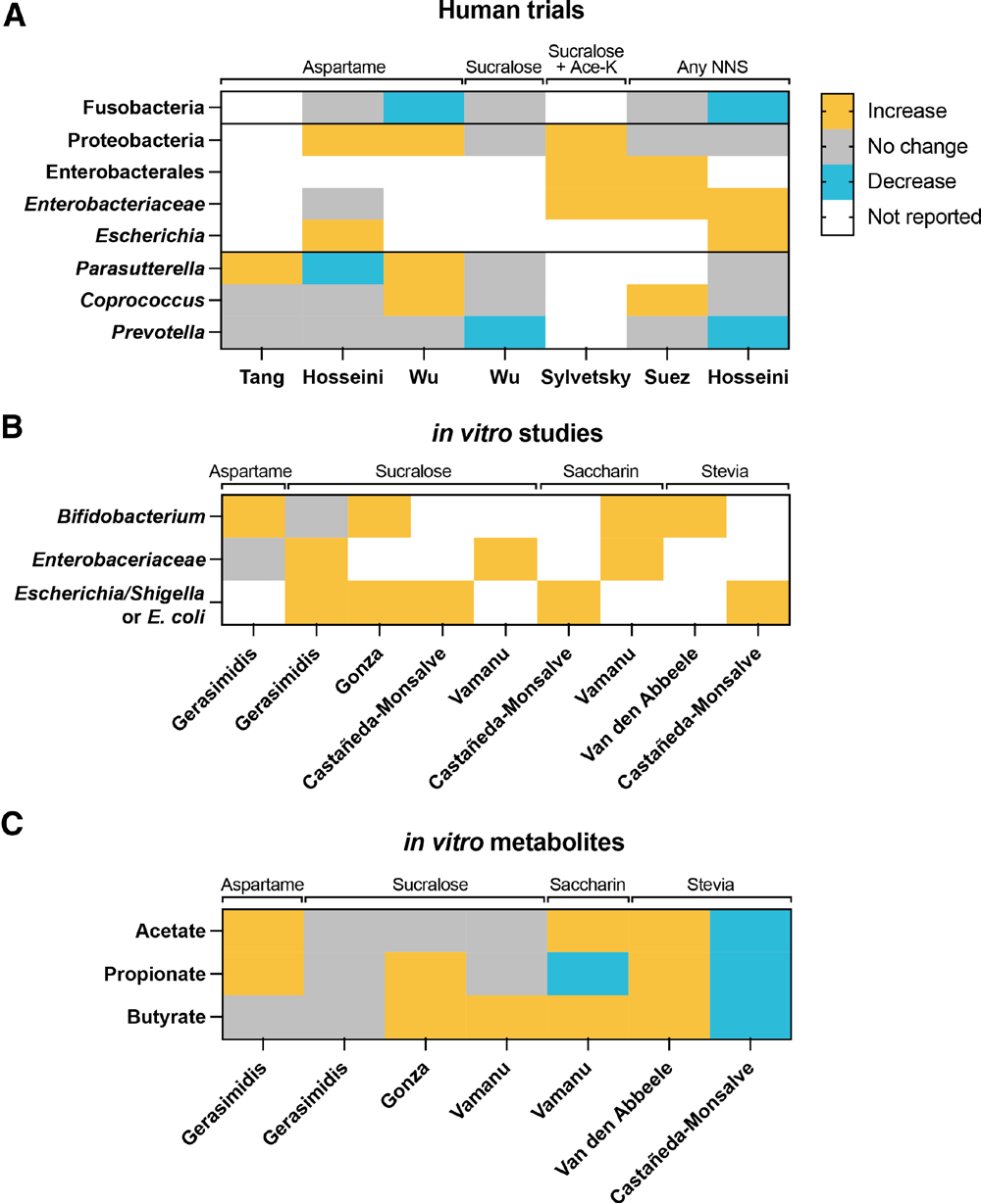
**Impact of non-nutritive sweeteners (NNS) on human gut microbiome and associated metabolites.** Statistically significant changes (following correction for multiple hypothesis testing) were observed in two or more trials. (A) Compositional changes in interventional and observational human trials. (B) Compositional and (C) metabolite changes observed in human-derived bacterial microbiome cultures. The first author’s names correspond to the studies listed in Tables 1 and 2.

Whether and how these NNS-associated microbiome signatures mediate the impacts of sweeteners on human health requires additional evidence. In addition to studies causally linking the microbiome of NNS consumers to metabolic effects in humanized gnotobiotic mice ^[32,33]^, identifying common taxonomic, functional, and metabolomic signatures across trials can shed light on the microbial features involved in mediating health effects of NNS. To that end, we sought to identify features reported to be impacted by sweeteners in the same direction (increased or decreased) in two or more of the aforementioned trials. Due to the limited available metagenomic and metabolomic data, we focused on taxonomic similarities. No individual taxonomic unit was reported in all trials, which is expected considering the heterogeneity of human microbiome composition. Nonetheless, several noteworthy patterns emerged (Figure 1A). Five studies reported a higher abundance of Proteobacteria and/or the phylogenetically related *Enterobacteriaceae* and *Escherichia* associated with NNS intake. No study reported a lower abundance of these taxa associated with NNS. Members of these taxonomic groups, particularly *Escherichia coli*, have been associated with type 2 diabetes in several human cohorts ^[63]^. A higher abundance of *Parasutterella*, another member of Proteobacteria previously associated with obesity and diabetes ^[64]^, was reported in two studies. However, a third study reported a lower abundance associated with NNS consumption. As most studies used microbiome profiling methods that do not provide species- and strain-level resolution (predominantly 16S sequencing), comparing the observed signatures to the literature is challenging. For example, two studies reported a higher abundance of the genus *Coprococcus* associated with NNS consumption, but *Coprococcus eutactus* has been previously associated with improved insulin sensitivity ^[63]^. Furthermore, two studies reported a lower abundance of *Prevotella* associated with NNS intake; specific clades of *Prevotella copri* have been associated with worsened metabolic health ^[63]^. Future studies should incorporate metagenomic profiling of the microbiome to facilitate comparisons across trials and the identification of the underlying mechanisms through which NNS impact health through the microbiome. Ultimately, linking any of the aforementioned taxonomic signatures to health impacts likely requires the use of preclinical models in which the metabolic health of animals administered with the bacteria of interest is monitored.

Studies culturing human gut-derived microbial communities with sweeteners can provide additional supporting evidence for the impact of NNS on the human microbiome. We found several studies in which NNS were added to microbiome communities derived from human stool samples and grown in bioreactors or batch cultures under conditions simulating the human gut ^[65–69]^. Using the aforementioned strategy, we sought to identify taxa and metabolites impacted in the same direction in two or more experimental arms (Figure 1B). Interestingly, the family *Enterobacteriaceae* and its genera *Escherichia-Shigella* (or the species *E. coli*) were significantly increased in five of six experimental arms that profiled these taxa ^[65,66,68]^; the sixth study, the only one with aspartame, reported no significant changes ^[65]^. The genus *Bifidobacterium* was increased in four of six experimental arms ^[65,67–69]^, and two found no significant differences ^[65]^. Several studies also profiled metabolites produced under these culture conditions. Of these, SCFA showed some consistent patterns, with several studies demonstrating increased levels of acetate, propionate, and butyrate following culturing with NNS, although opposite results were also reported (Figure 1C). Interestingly, the increase in members of Proteobacteria and specifically *Enterobacteriaceae* is in line with the human interventional/observational studies (Figure 1A) as well as results in preclinical trials ^[12]^. It also aligns with the observation that *Enterobacteriaceae* are commonly associated with type 2 diabetes in humans ^[70,71]^. The increase in acetate and propionate also aligns with previous findings in NNS-fed animals ^[12]^, although the association between SCFA and metabolic health is more complex. Elevated levels of SCFA have been observed in mice ^[72]^ and humans ^[73]^ with obesity; diets rich in saturated fat were associated with MetS and elevated SCFA levels in humans ^[74]^ and mice ^[75]^; and weight loss in humans was associated with reduced plasma propionate ^[76]^. In contrast, supplementation with either acetate, propionate, or butyrate is generally associated with improved metabolic markers in both humans ^[77,78]^ and preclinical models ^[79–82]^. In the context of NNS, additional research is required to determine whether the elevated SCFA levels contribute to detrimental metabolic effects or are simply byproducts of broader microbiome modulation. However, as not all studies that measure SCFA find higher quantities associated with NNS, they are likely not the exclusive mechanism through which the NNS-modulated microbiome impacts metabolic health.

These ex vivo results should be interpreted with care, as the dose added to the microbial cultures can be markedly different than that found in the gut. Furthermore, ex vivo microbial communities tend to differ in composition from their original source, as some bacteria require growth conditions that are difficult to replicate outside of the gut. Simulating the gut environment in culture requires consideration of the growth medium, as the impact of NNS on bacteria might depend on the availability of other nutrients. Additional factors that can impact the results, such as regulation of pH and metabolite influx, can be controlled using bioreactors. Finally, some impacts of NNS on gut bacteria could depend on the crosstalk between the microbiome and the host and cannot be replicated in these ex vivo studies. Inherently, this type of experiment cannot determine whether NNS-induced changes to the microbial community are beneficial, neutral, or detrimental to metabolic health. Nonetheless, this type of evidence can support findings in human interventional and observational trials. Furthermore, it can facilitate the discovery of the mechanisms through which NNS impact the microbiome.

Taken together, evidence from interventional and observational trials in humans, as well as cultures of human gut-derived microbial communities with NNS, support the notion that NNS can alter the human microbiome and, consequently, metabolic health. The human microbiome may be more resilient to the impact of NNS than that of laboratory animals, although the discrepancy between preclinical and clinical trials can merely reflect human microbiome heterogeneity. Additional trials are critically needed to identify a consistent signal of NNS impacts on the human microbiome, as well as the methodological and/or biological factors that contribute to the variation between trials.

## 4. Towards a precision nutrition approach to NNS

Precision and personalized medicine seek to utilize patient-specific parameters to tailor and adjust therapeutics to improve clinical efficacy and reduce adverse effects. The human microbiome plays a key role in numerous aspects of human health and presents considerable person-to-person variation, making it an attractive component to consider when devising precision therapeutics ^[83]^. As a notable example, the microbiome has been shown to mediate clinical responsiveness to anti-cancer immunotherapies ^[84–86]^, which can be improved upon microbiome modulation using fecal microbiome transplantations from clinical responders or healthy donors ^[87–90]^.

Using a similar framework to precision medicine, precision nutrition seeks to tailor dietary recommendations to the individual to improve their health, often emphasizing cardiometabolic health parameters. Several studies have reported that a response to a weight-loss diet can be predicted according to the microbiome composition ^[91]^, function ^[92]^, or diversity ^[93]^, and similarly, the microbiome can predict the response to a diet aimed at improving glucose metabolism ^[94]^. Microbiome features are also increasingly studied for their ability to predict post-prandial glycemic responses. While dietary recommendations focus on the food’s properties, such as its carbohydrate content ^[16]^, considerable person-to-person variation has been observed in post-prandial glycemic and lipidemic responses to identical foods ^[95–97]^, limiting the applicability of population-level dietary recommendations to improve an individual’s health. Considering person-specific features, including the gut microbiome, can significantly improve our ability to predict post-prandial metabolic responses to any given food item ^[96–99]^. Based on these discoveries, several studies have demonstrated that a personalized, microbiome-based, and algorithm-devised diet outperforms standard-of-care diets in patients with prediabetes ^[100]^ or type 2 diabetes ^[101]^.

Considering NNS in the context of precision nutrition is compelling, as it can assist in resolving the conflicting outcomes of RCTs examining NNS impacts on cardiometabolic health. If NNS are detrimental to metabolic health in some individuals, but neutral or beneficial in others, then the outcome of any RCT will depend on the fraction of enrolled responders (detrimental/beneficial) vs non-responders. The extent to which the impact of NNS on metabolic health varies between individuals and the underlying mechanisms are currently underexplored. The first interventional study that reported an effect of NNS on the human microbiome (*N* = 7) identified that saccharin impaired glucose tolerance in a subset of supplemented individuals, and the authors noted that both the baseline microbiome composition and the extent to which it changed during saccharin supplementation differed between responders and non-responders. Through a series of fecal microbiome transplantation experiments in GF mice, the authors observed that the post-saccharin exposure microbiome of responders elicited a poorer glycemic response compared with the pre-saccharin exposure communities from the same individuals. In contrast, pre- and post-saccharin exposure microbiomes from non-responders elicited comparable glycemic responses in GF mice ^[32]^. In a follow-up study (*N* = 120), the authors reported that sucralose and saccharin disrupt glucose tolerance, although the impact varied between individuals in these two groups, as well as in two additional NNS groups (aspartame and stevia). There were notable differences in how each NNS impacted the microbiome composition and function of top and bottom responders. Again, the authors used extensive transplantations to GF mice to establish a causal link between the microbiome and the extent to which glucose homeostasis was disrupted. The post-exposure microbiome from top responders in all four NNS groups, but not the control groups, resulted in a poorer glycemic response than that elicited by the pre-exposure microbiome of the same individuals ^[33]^.

How the microbiome dictates the extent to which NNS impact glucose tolerance (and potentially other metabolic effects) in humans is currently unknown. However, the diverging microbiome profiles of responders and non-responders to NNS, both before and after supplementation ^[32,33]^, suggest that some gut bacteria are more resistant to NNS than others. Thus, different microbiome configurations can vary in their amenability to NNS-induced alterations. In line with this hypothesis, an in vitro study that simulated the impact of various food additives on human gut bacteria batch cultures found differing and sometimes opposing effects of sucralose on the microbiome derived from patients with IBD vs the microbiome of controls ^[68]^. Future human trials should incorporate stratification of participants based on their metabolic response to NNS and examine the correlations to the microbiome’s trajectory during the trial. Additional large-scale trials are required to identify microbial markers that can predict individualized responses to NNS.

## 5. NNS may disrupt metabolic health through multiple mechanisms

The human trials described earlier, coupled with experiments in animal models, provide compelling evidence for a link between NNS-induced microbiome alterations and disruption of metabolic health. Several studies further show that these microbiome alterations are sufficient to impact glucose tolerance ^[32–34]^ and weight ^[34]^ in NNS-naive GF mice. The mechanisms through which the NNS-altered microbiome impacts the metabolic health of the recipient GF mice are underexplored; in one study, poorer glycemic response in the recipient mice was associated with a reduced bacterial capacity for metabolizing dietary or host-derived carbohydrates ^[33]^. Studies in animals directly exposed to NNS have reported co-occurrence of microbiome shifts and metabolic derangements with several physiological alterations that can hint at the underlying mechanisms. However, such studies do not demonstrate that the microbiome is the cause, rather than an additional outcome, of these alterations. Among these alterations, NNS supplementation has been associated in animal models with the induction of systemic or localized inflammation in the gut or metabolic tissues ^[102–108]^. Coupled with studies associating NNS with an increased abundance of bacterial lipopolysaccharide ^[12]^ and impaired intestinal barrier function ^[104,107,109]^, sweeteners may exacerbate metabolic endotoxemia ^[110]^.

In parallel, multiple studies have explored the physiological impacts of NNS supplementation without addressing the involvement of the microbiome in these processes, including receptor-mediated taste preferences, neurometabolic signaling, and metabolic reprogramming.

### 5.1 Taste preferences

Functional testing in humans has revealed that sucralose activates G protein-coupled taste receptors T1R2 and T1R3 on the tongue ^[111,112]^, signaling brain regions associated with food reward, such as the frontal operculum/anterior insula ^[113]^. The binding affinity of sweeteners to these receptors appears to increase with sweetness intensity, with distinct patterns observed for different NNS like advantame, sucralose, and saccharin, compared with sugar alcohols. This suggests that NNS consumption could have long-lasting effects on sweet preference, sugar cross-adaption, or desensitization, potentially influencing metabolic outcomes even in early life, including in utero ^[114–116]^.

### 5.2 Incretins

Sweet taste receptors also exist in extra-oral tissues, such as the gut and pancreas, where they modulate glucose absorption and hormone secretion, including glucagon-like peptide-1 (GLP-1) and gastric inhibitory polypeptide (GIP). However, the effects of NNS on these processes are inconsistent across species. While rodent studies have shown enhanced GLP-1 and GIP release following NNS exposure ^[117,118]^, human trials have yielded mixed results, with some showing no significant effects ^[119–122]^.

### 5.3 Uncoupling

NNS may disrupt cognitive pathways, potentially decoupling sweet taste from caloric content, which could impair cephalic phase responses responsible for anticipatory physiological reactions that prepare the body for nutrient absorption, thereby disrupting energy regulation and increasing obesity risk. The absence of a cephalic phase insulin response has been linked to impaired insulin secretion and an elevated risk of obesity. However, these effects vary by sweetener and are not consistently observed ^[123,124]^.

Collectively, these studies suggest that NNS are not metabolically inert and may have complex, multifaceted effects on energy balance, food intake, and overall metabolic health. As indicated earlier, NNS-induced microbiome alterations are sufficient to disrupt metabolic health, though many of the mechanistic aforementioned studies did not address the role of the microbiome. Some of these mechanisms could be a downstream result of microbiome alterations (eg, immune signaling). Alternatively, as some of these mechanisms have been demonstrated in sterile cell culture, NNS likely impact health through several unrelated microbiome-dependent and independent mechanisms, although additional evidence in humans is needed.

## 6. Conclusions and perspective

The WHO meta-analysis and conditional recommendation against the use of NNS for the prevention and treatment of weight gain and diet-related non-communicable diseases highlighted the critical importance of obtaining more evidence causally linking NNS consumption to impacts on human health. Given the variability in published trials regarding NNS safety, accumulating more data using existing trial designs may not resolve this public health issue. A personalized nutrition perspective in RCTs, using predetermined criteria for participant stratification according to observed metabolic effects, can help identify the prevalence of susceptibility to NNS-related health impacts and their underlying causes. In that context, the person-to-person heterogeneity of the human microbiome, coupled with its critical roles in cardiometabolic health, makes it an important target to consider as a personalized modulator of NNS impacts on health. Notably, while the WHO recommendation applies to individuals of all ages, including pregnant and lactating individuals, it does not apply to those with pre-existing diabetes because the assessment of health outcomes to inform disease management was beyond the scope of the guideline. Considering the prevalence of NNS consumption in this population, it is critical to extend the scope of research on NNS-microbiome interactions beyond trials with normoglycemic populations.

As summarized, several trials have reported significant impacts of various NNS on the human gut microbiome. However, the impact of some sweeteners, such as allulose and monk fruit, remains completely unknown. Across trials, a significant alteration of the microbiome by NNS was correlated with a detrimental impact on metabolic health. The trials that found no significant impact of NNS on the microbiome were also the ones in which NNS did not impact metabolic health. This observation, coupled with trials that addressed personalization directly ^[32,33]^, supports that the microbiome plays a role in mediating personalized susceptibility to the metabolic impacts of NNS. It is therefore recommended that future RCTs on the metabolic impacts of NNS will include microbiome profiling, preferably using shotgun metagenomics, as using 16S sequencing was more common in trials that did not find a microbiome signature, potentially due to human microbiome compositional heterogeneity. Two additional parameters that are correlated with the impact of NNS on the microbiome are the formulation in which they are supplemented (pure or with a carbohydrate) and the extent to which participants were naïve to sweeteners at baseline. The contribution of these two parameters to the heterogeneity between trials is insufficiently clear. Preclinical trials could be used to address this question and inform the design of future clinical trials.

Two of the studies covered in this analysis included a unique component that should be considered in future clinical trials. Sylvetsky et al ^[43]^ quantified the supplemented sucralose in the participants’ urine to determine adherence. This approach could also be used to correlate personalized differences with the amount of sweetener found in urine and feces. Methods that can simultaneously quantify multiple NNS in a sample ^[47]^ would be able to account not only for adherence to the supplemented product but also for avoidance of other NNS-containing products. In an observational trial, Hosseini et al ^[57]^ found that NNS were associated with diverging effects on the duodenal and fecal microbiome. The extent to which different NNS impact the small intestine microbiome and its importance in mediating their impacts on health is currently unclear. Here again, preclinical models can be utilized to address this question, as direct in situ sampling of the microbiome remains challenging to execute on a large scale.

Identifying the microbial features (specific species, functions, or metabolites) that mediate the impact of each NNS on metabolic health requires more data. We were able to identify a few microbial patterns that were common to several trials, though none were consistent across all trials. Notably, our approach was simplistic and relied on the identification of microbial taxa by name. It would be interesting to reanalyze all the available datasets from the aforementioned trials as part of a meta-analysis on the impact of NNS on the human gut microbiome, as was previously done for high-fat diet in mice ^[35]^ and with the help of tools that can harmonize 16S and metagenomic datasets ^[125]^.

Notably, in two of the observational trials that provided associations between NNS intake and an impact on the microbiome, NNS were not the focus of the study but rather reported as part of the participants’ dietary components ^[54,55]^. With the expansion of large-scale citizen science initiatives like the American Gut Project ^[126]^ and the Human Phenotype Project ^[127]^ that collect both microbiome and dietary data, it would be valuable to include specific dietary questions on the intake of NNS-containing products and harness these large datasets to detect not only associations between NNS and the microbiome but also potential modulators of their interaction. However, data on the impacts of NNS on the microbiome stem almost exclusively from North America and Western Europe (Tables 1 and 2), with currently no information available on populations living in Africa, Australia, South America, Eastern Europe, or East Asia. While this under-representation is not unique to the field of NNS ^[128]^, it would be valuable to encourage initiatives that increase the representation of under-studied populations in trials dissecting the impacts of NNS on the microbiome and metabolic health.

## Conflicts of interest

The authors declare that they have no conflicts of interest.

## Funding

K.R.C. is supported by the National Institutes of Health grant T32 OD011089. J.S. is the inaugural Feinstone Assistant Professor in the W. Harry Feinstone Department of Molecular Microbiology and Immunology at the Johns Hopkins Bloomberg School of Public Health. Research in the Suez lab is supported by the National Institutes of Health, Office of the Director, under Award Number DP5OD029603. The content is solely the responsibility of the authors and does not necessarily represent the official views of the National Institutes of Health.

## Acknowledgments

The authors thank the members of the Suez lab for fruitful discussions.
